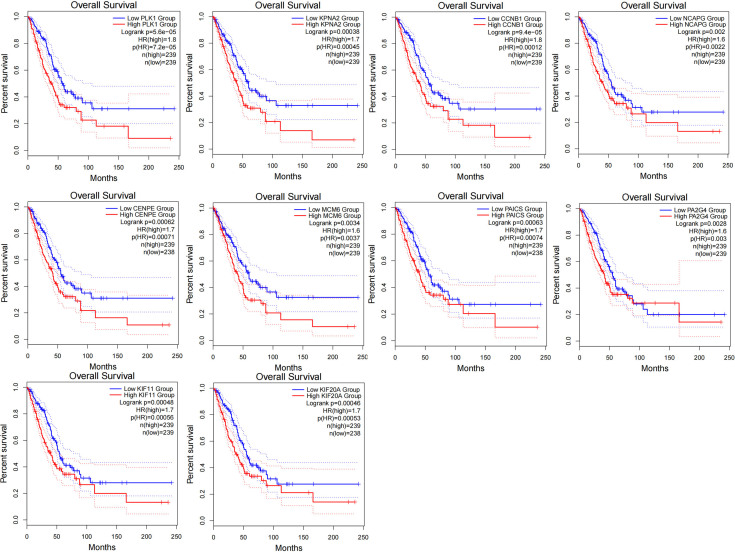# Chaperonin-containing TCP-1 subunit genes are potential prognostic biomarkers and are correlated with Th2 cell infiltration in lung adenocarcinoma: An observational study

**DOI:** 10.1097/MD.0000000000038387

**Published:** 2024-05-31

**Authors:** Ruijuan Du, Zijun Zhou, Yunlong Huang, Kai Li, Kelei Guo, Li Han, Hua Bian

**Affiliations:** a Zhang Zhongjing School of Chinese Medicine, Nanyang Institute of Technology, Nanyang, Henan Province, PR China; b Henan Key Laboratory of Zhang Zhongjing Formulae and Herbs for Immunoregulation, Nanyang Institute of Technology, Nanyang, Henan Province, PR China.

**Keywords:** bioinformatics, biomarker, CCTs, immune cell infiltration, lung adenocarcinoma

## Abstract

A family of molecular chaperone complexes called chaperonin-containing T-complex protein 1 (TCP-1) subunit genes (CCTs) aids in the folding of numerous proteins. With regard to lung adenocarcinoma (LUAD), this study provided a thorough understanding of the diagnostic and prognostic use of CCTs. The expression of CCTs in LUAD was evaluated by using databases including UALCAN and the Gene Expression Omnibus. Immunohistochemistry (IHC) was conducted to validate the expression of CCTs in LUAD. The mutation in the CCTs was identified through the cBioPortal database, while promoter methylation was measured by the UALCAN database. The prognostic value of CCTs was evaluated using the PrognoScan analysis. The GEPIA2.0 database was used to measure the prognostic value of CCTs and associated Hub genes. Correlation analysis between CCTs expression in LUAD was based on the GEPIA2.0 database. The ROC curves, clinical correlation analysis, gene ontology, Kyoto Encyclopedia of Genes and Genome analysis, and immune cell infiltration analysis were downloaded from The Cancer Genome Atlas database and then analyzed and visualized using the R language. The STRING database was used for protein–protein interaction analysis. Upregulation of CCTs expression in patients with LUAD indicated advanced diseases and a poor prognosis. ROC curve analysis revealed that the CCTs may serve as diagnostic indicators. The functional enrichment analysis showed that CCTs were involved in the mitosis-mediated cell cycle process. Additionally, 10 hub genes associated with CCTs that were linked to LUAD prognosis and tumor progression were identified. Immune cell infiltration analysis showed that CCTs expression in tumor tissues tends to be related to T helper type 2 cell infiltration. This study revealed that CCTs may serve as valuable biomarkers for the diagnosis and targeted therapy of LUAD.

## 1. Introduction

Lung cancer is one of the most commonly diagnosed malignancies and accounts for most cancer-related deaths globally. Approximately 2.20 million new cases and 1.79 million deaths occur annually due to lung cancer.^[1]^ Lung cancer outcomes have remained poor in recent years despite a decline in the mortality rate.^[1]^ Non-small-cell lung cancer (NSCLC) comprises approximately 85% of all subtypes of lung cancer.^[2]^ The two most prevalent histological subtypes are lung squamous cell carcinoma (LUSC) and lung adenocarcinoma (LUAD), with the latter accounting for approximately 40% to 50% of all NSCLC.^[2,3]^ Although smoking is associated with all main subtypes of NSCLC, the correlation is stronger for LUSC than for LUAD, given that LUAD is the most commonly observed histology in individuals who have never smoked.^[4]^ Moreover, LUAD is more common in women and is associated with secondhand smoking, environmental pollution, and genetic susceptibility.^[5,6]^ Mutations in genes such as EGFR, MET, and ALK are detected in LUAD and targeted therapies are beneficial for these patients.^[7]^ Additionally, oncogenic pathways and the tumor microenvironment are research hotspots, and some developments have been implemented in clinical settings.^[7]^ However, a large proportion of patients with LUAD continue to exhibit inadequate responses to current therapies. Identification of new biomarkers for LUAD patients and exploration of more effective therapeutics remain areas of unmet need for patients with LUAD.

This study focused on chaperonin-containing TCP-1 (T-complex protein 1) subunit genes (CCTs), a molecular chaperone complex that aids in the folding of proteins reliant on ATP hydrolysis, to progress the identification of targetable factors for LUAD therapy.^[8]^ CCTs consist of the following subunits: TCP1, CCT2, CCT3, CCT4, CCT5, CCT6A, CCT6B, CCT7, and CCT8. The domains of the CCTs protein structure include the apical domain, responsible for substrate binding; the hinge domain, which connects to the apical domain; the equatorial domain, which constitutes the base of the chamber; and the intermediate domain, which contains the ATP-binding pocket.^[9]^ According to the CCTs interactome, CCTs may interact with various proteins associated with cytoskeletal function, chromatin remodeling, secretory pathways, phosphatase activity, and cell cycle regulation.^[10]^ Based on computational analysis, CCTs-interacted proteins have been identified to be involved in shock, stress, HIV infections, immunologic deficiency syndromes, and cancer.^[11]^

The biological functions of CCTs and their interacted proteins suggest their potential involvement in various human diseases, including cancer. CCTs have been reported to function as oncogenes in several human cancers, including breast cancer,^[12–14]^ laryngeal squamous cell cancer,^[15]^ esophageal squamous cell carcinoma,^[16]^ metastatic LUAD,^[17]^ thyroid neoplasia,^[18]^ and hepatocellular carcinoma.^[19–21]^ However, there is a lack of systematic, thorough analysis and understanding of CCTs in LUAD. Therefore, this study aimed to provide a comprehensive investigation of CCTs in LUAD, including an assessment of their expression, clinical significance, molecular mechanism, and immunity correlation analyses.

In the current study, the expression of CCTs was significantly elevated in LUAD tumor tissues compared with that in normal tissues. A higher level of CCTs expression in LUAD patients was associated with an advanced clinical stage and a poor prognosis. Additionally, this study demonstrated that CCTs were involved in cell cycle progression mediated by mitosis and T helper type 2 (Th2) cell infiltration in the tumor microenvironment. A comprehensive evaluation of CCTs in LUAD was performed using several public platforms and bioinformatics analysis, which provided evidence that CCTs may serve as beneficial indicators for the diagnosis of LUAD as well as personalized treatment.

## 2. Methods

### 2.1. Patients

The immunohistochemistry (IHC) assay included 30 patients who underwent partial pneumoresection with the approval of the Institutional Ethics Committee of Shanghai Outdo Biotech Company. The inclusion criteria of patients were as follows: (I) postoperative pathology determined the presence of LUAD; (II) most patient’s medical records were available; (III) no comprehensive anti-tumor therapies, such as immunotherapy, chemotherapy, targeted therapy, or radiotherapy, were performed before surgery. The exclusion criteria of patients were as follows: (I) those who had any additional forms of cancerous tumors and (II) those whose malignancies have metastasized from other cancerous tumors. This study was performed with the approval of the Institutional Ethics Committee of Shanghai Outdo Biotech Company [Approval number: SHYJS-CP-1904007]. Written informed consent was provided by all participants before undergoing surgical treatment.

The mRNA expression of CCTs was evaluated in 535 LUAD tissues and 59 normal lung tissues obtained from The Cancer Genome Atlas (TCGA) database. A total of 175 (32.9%) cases were classified as stage T1, 289 (54.3%) as T2, 49 (9.2%) as T3, and 19 (3.6%) as T4. Stage N0 accounted for 348 (67.0%) of the cases, N1 for 95 (18.3%), N2 for 74 (14.3%), and N3 for 2 (0.4%). Stage M0 was assigned to 361 (93.5%) patients, whereas stage M1 was assigned to 25 (6.5%) patients. Clinical characteristics and associated gene data of LUAD patients were also downloaded.

### 2.2. Reagents

Anti-TCP1 (#R27336) and anti-CCT2 (#R26528) antibodies were purchased from Zenbio Science (Chengdu, China). Anti-CCT3 (#10571-1-AP), anti-CCT4 (#21524-1-AP), anti-CCT7 (#15994-1-AP), and anti-CCT4 (#67539-1-Ig) antibodies were purchased from Proteintech Group. Anti-CCT5 (#D125930) and anti-CCT6A (#D153384) antibodies were purchased from Sangon Biotech (Shanghai, China).

### 2.3. Immunohistochemistry assay

The Shanghai Outdo Biotech Company (Shanghai, China) provided the human LUAD tissue arrays. For antigen retrieval, the samples were boiled in sodium citrate buffer (10 mmol/L, pH 6.0) for 10 minutes. The samples were then exposed to 3% H_2_O_2_ for 10 minutes. Each slide was incubated with 10% goat serum albumin at room temperature for 1 hour in a humidified environment. Subsequently, the slides were left to be treated with primary antibodies overnight at 4 °C. The following day, the secondary antibody was added to the slides and incubated for 30 minutes at room temperature. The sections were finally counterstained with hematoxylin, dehydrated, covered, and visualized. The study was conducted in accordance with the Declaration of Helsinki (as revised in 2013).

### 2.4. UALCAN analysis

To analyze cancer OMICS, the online UALCAN database (http://ualcan.path.uab.edu) was based on data from the TCGA.^[22]^ In this study, we compared the mRNA and protein expression of CCTs between LUAD normal and tumor samples using UALCAN. Additionally, promoter methylation of CCTs in LUAD between normal and tumor samples was also obtained from the UALCAN database. Expression levels of CCTs in LUAD were compared using Student *t* test, and *P* < .05 was considered statistically significant.

### 2.5. TIMER2.0 analysis

The TIMER2.0 database, which is accessible at http://timer.cistrome.org, is a comprehensive resource for systematic investigation of the relationships between immune infiltrates and gene expression, differences in gene expression between tumors and normal tissues, and estimates of immune infiltration.^[23,24]^ The mRNA expression and mutation rate of CCTs were evaluated in various types of cancers using the TIMER2.0 database. The expression levels of CCTs in normal and tumor tissues were compared using Student *t* test, and *P* < .05 was considered statistically significant.

### 2.6. GEPIA2.0 analysis

The GEPIA2.0 database (http://gepia2.cancer-pku.cn/#index) offers several features, including patient survival analysis, profiling based on cancer types or pathological stages, differential expression in cancer, correlation analysis between different genes, and dimensionality reduction analysis. This study used the GEPIA2.0 database to analyze the predictive significance of CCTs and their associated Hub genes in LUAD patients. Additionally, the GEPIA2.0 database served as the basis for the correlation analysis between the expression of different CCTs in LUAD. The association between the expression of CCTs and their co-expressed genes was investigated using the GEPIA2.0 database. A person-correlation analysis was used, and *P* < .05 was considered statistically significant.

### 2.7. PrognoScan analysis

The PrognoScan database (http://www.prognoscan.org/) is a newly developed database to facilitate meta-analysis of the prognostic value of genes. Using various publicly available cancer microarray datasets, this study elucidated the relationship between gene expression and patient’s prognosis, including overall survival (OS) and disease-free survival (DFS).^[25]^ PrognoScan was used to examine the correlation between CCTs expression and OS (GSE13213) or DFS (GSE31210) of LUAD patients, along with the prognostic value of CCTs mRNA expression. The LUAD patients were categorized into high and low CCTs expression groups and were examined using a Kaplan–Meier survival plot, which exhibited a hazard ratio with 95% confidence intervals.

### 2.8. cBioPortal analysis

Access to multidimensional cancer genomics data, including somatic mutations, alterations in DNA copy number, mRNA and miRNA expression, DNA methylation, and protein abundance, is available through the online cBioPortal database (www.cbioportal.org).^[26]^ The mutations of CCTs in LUAD patients and the association between CCTs mutation and LUAD patient survival were assessed using cBioPortal. Kaplan–Meier analysis was designed to evaluate the relationship between CCTs mutation and OS or DFS in LUAD patients. Log-rank test was utilized, and *P* < .05 was considered to be statistically significant.

### 2.9. Protein–protein interaction analysis

The STRING database (https://string-db.org/) was used to assess the direct and indirect correlations between known and expected protein–protein interactions. These interactions arise from knowledge transfer between species, computer prediction, and interactions compiled from other databases.^[27]^ Potential interactions between CCTs subunits and between CCTs and their co-expressed genes were investigated using the STRING database. After downloading the protein–protein interaction data from STRING, Cytoscape 3.6.1 software was used to analyze and visualize the data. The top 10 genes were classified as Hub genes using the CytoHubba plug-in as the Hub gene screening standard.

### 2.10. GEO database analysis

The GSE32863 dataset was downloaded from the Gene Expression Omnibus (GEO) website (https://www.ncbi.nlm.nih.gov/geo/query/acc.cgi?acc=GSE32863). It exhibited expression profiles of 58 LUAD and 58 adjacent non-tumor lung fresh frozen tissues. CCTs expression profiling was extracted from the downloaded data and analyzed using GraphPad Prism 6.

### 2.11. TCGA data analysis

After obtaining the RNA sequencing and associated clinical data from the TCGA database, R software (Version 3.6.3; https://www.Rproject.org) was used to analyze and visualize the data. The R package “ggplot2” was used to visualize most of the analyzed data; however, the chord chart was visualized using the “circlize” package. For the ROC curves, the “pROC” package was used for data analysis. Gene ontology and Kyoto Encyclopedia of Genes and Genome analyses were performed on CCTs-co-expressed genes with the package R “clusterProfiler” for enrichment analysis and the package R “org.Hs.e.g..db” for ID transformation. Immune cell infiltration analysis was executed using “GSVA” and “ggplot2” R packages. Correlation analysis of CCTs and immune-related genes was performed using the TCGA database and visualized using the R “ggplot2” package.

### 2.12. Statistical analysis

SPSS v.19 software was used for statistical analyses. Student *t* test was used to assess the statistical differences between the paired and unpaired samples. Survival analysis was conducted between the high- and low-risk groups using Kaplan–Meier analysis. Pearson test was used to investigate the correlation between the two variables. Cox regression analysis was used to univariately and multivariately analyze CCTs and clinical features. The *P* < .05 was considered to be statistically significant.

## 3. Results

### 3.1. Expression of CCTs in various cancer types

The mRNA expression levels of CCTs across a variety of cancer types were assessed using the TIMER2.0 database. The data revealed that CCTs expression was generally upregulated in most cancers, except for CCT6B (Fig. S1, Supplemental Digital Content, http://links.lww.com/MD/M700 and Table S1, Supplemental Digital Content, http://links.lww.com/MD/M708). The expression of all 8 CCT members was increased in cholangiocarcinoma, colon adenocarcinoma, esophageal carcinoma, head and neck squamous cell carcinoma, liver hepatocellular carcinoma, LUAD, LUSC, prostate adenocarcinoma, rectum adenocarcinoma, and stomach adenocarcinoma, except for CCT6B. Additionally, the link between CCTs expression and patient survival was explored. The GEPIA2.0 database revealed that the high expression of CCTs in cancer tissues, except for CCT6B, predicted a poor prognosis for patients with liver hepatocellular carcinoma and LUAD (Table S2, Supplemental Digital Content, http://links.lww.com/MD/M709). Interestingly, except for CCT6B, all eight members of the CCTs with high expression exhibited a poor prognosis in LUAD patients rather than LUSC. Therefore, a comprehensive analysis of eight members of the CCTs family, including TCP1, CCT2, CCT3, CCT4, CCT5, CCT6A, CCT7, and CCT8, was performed to further investigate the clinical importance and molecular mechanism of CCTs in LUAD.

### 3.2. CCTs expression in LUAD

**Figure 1.**
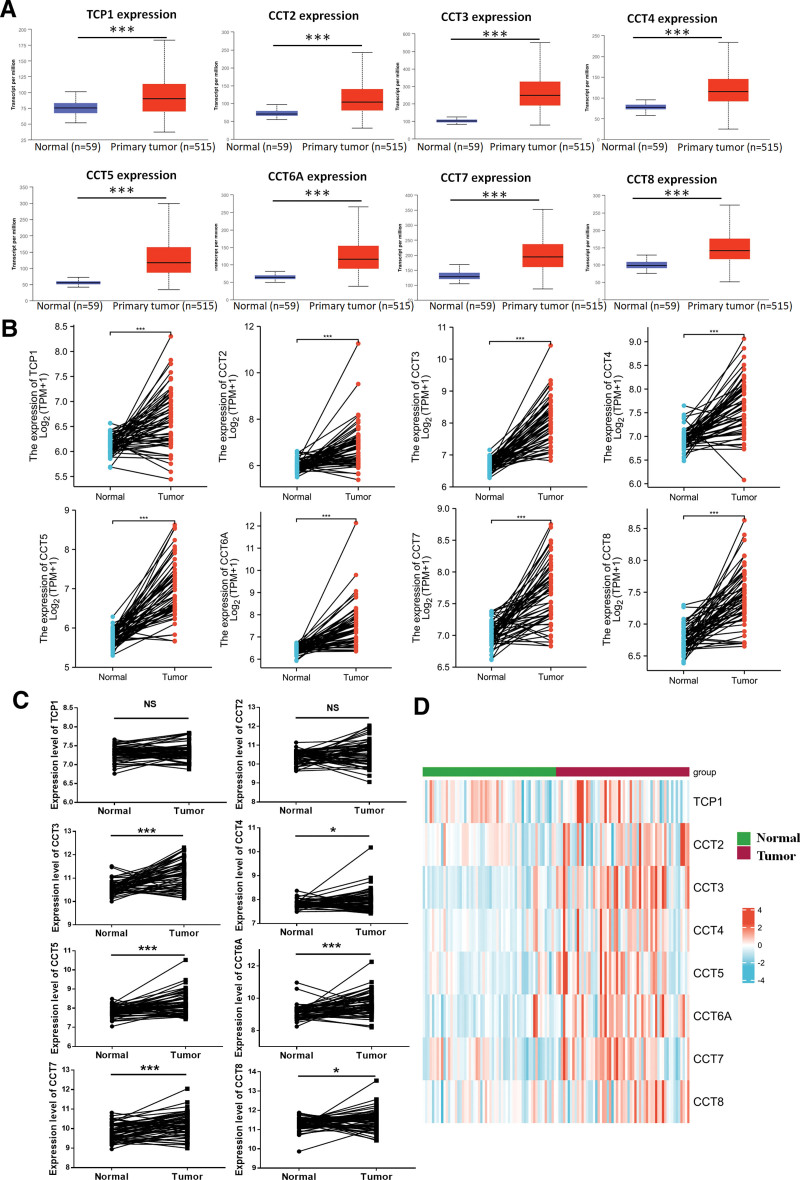
mRNA expression of CCTs in LUAD patients. (A) Expression of CCTs in normal lung tissues and LUAD tumor tissues was evaluated using the UALCAN database. (B) Expression of CCTs in normal lung tissues and paired LUAD tumor tissues was measured based on the TCGA database. (C) Expression of CCTs in 58 paired normal lung tissues and LUAD tumor tissues was measured based on the GEO database (GSE32863). (D) The heatmap of CCTs expression from GSE32863 data. *P < .05, ***P < .001. CCTs = chaperonin-containing TCP-1 (T-complex protein 1) subunit genes, LUAD = lung adenocarcinoma, TCGA = The Cancer Genome Atlas.

**Figure 2.**
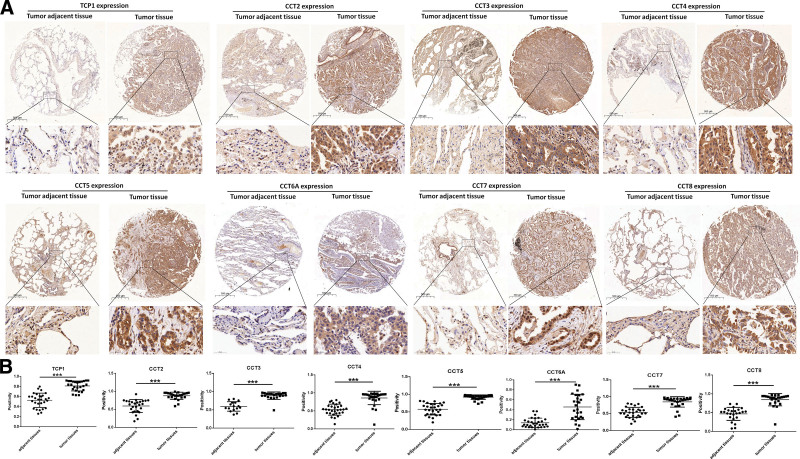
Protein expression of CCTs in LUAD patients. (A) The immunohistochemistry staining images of CCTs in tumor adjacent tissues and LUAD tumor tissues. (B) The statistical analysis of CCTs protein expression. ***P < .001. CCTs = chaperonin-containing TCP-1 (T-complex protein 1) subunit genes, LUAD = lung adenocarcinoma.

We initially explored CCTs expression in LUAD at the mRNA level using the UALCAN database. The results showed that the expression level of the CCTs family was significantly higher in LUAD tumor tissues than in normal tissues, with a *P*-value <.001 (Fig. 1A). The CCTs expression was further analyzed in paired LUAD tumor tissues compared with normal tissues based on TCGA data. Significant upregulation of CCTs was observed in LUAD tumor tissues compared with paired normal tissues (Fig. 1B). To further validate this result, we analyzed CCTs expression using GEO database. According to GSE32863 sequencing data, increased expression of CCT3, CCT4, CCT5, CCT6A, CCT7, and CCT8 was observed in LUAD tumor tissues compared with paired normal tissues (Fig. 1C). A heatmap of CCTs expression from GSE32863 is displayed in Figure 1D. Then, CCTs expression at protein levels was also measured using an IHC assay. The images showed stronger staining of CCTs in tumor tissues than in adjacent tumor tissues (Fig. 2A). Staining revealed that all CCTs exhibited cytoplasmic localization (Fig. 2A). Furthermore, TCP1, CCT2, CCT5, and CCT7 also showed strong nuclear expression (Fig. 2A). Statistical analysis revealed significantly increased protein levels of CCTs in tumor tissues compared with those in adjacent tumor tissues (Fig. 2B). Therefore, patients with LUAD exhibited widespread upregulation of CCTs at both the mRNA and protein levels.

### 3.3. Mutation and methylation of CCTs in LUAD

**Figure 3.**
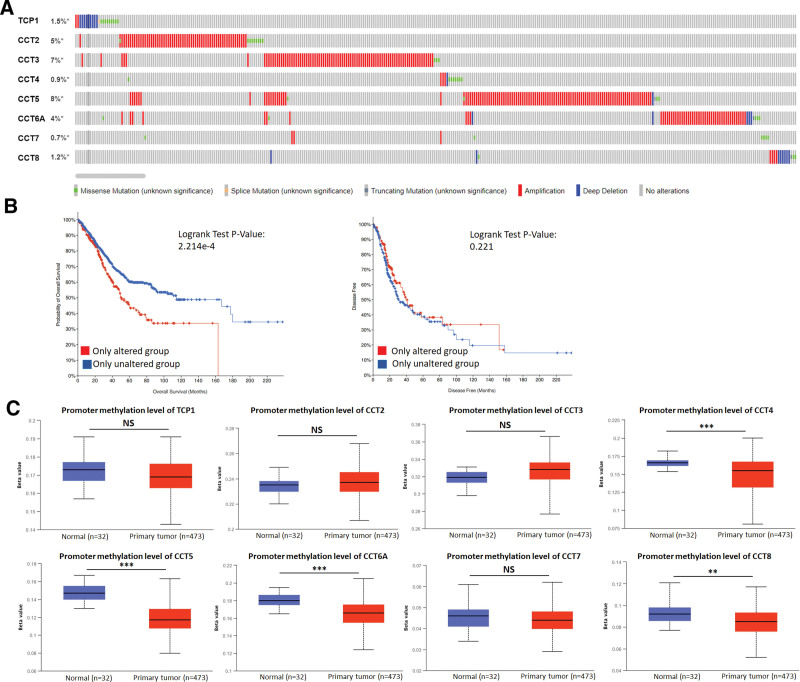
Mutation and methylation of CCTs in LUAD. (A) Mutations of CCTs in LUAD according to cBioPortal database. (B) Correlation between CCTs mutation and survival of LUAD patients. (C) Methylation of CCTs in LUAD based on the UALCAN database. **P < .01, ***P < .001. CCTs = chaperonin-containing TCP-1 (T-complex protein 1) subunit genes, LUAD = lung adenocarcinoma.

The TIMER2.0 database was used to identify CCTs mutation status across various tumor types. All 8 CCTs showed the highest mutation rate in uterine corpus endometrial carcinoma (Fig. S2, Supplemental Digital Content, http://links.lww.com/MD/M701). CCTs mutations and copy number alterations were then investigated in LUAD using the cBioPortal database. The mutation rate for CCT2 (5%), CCT3 (7%), CCT5 (8%), and CCT6A (4%) was relatively higher in LUAD than that for TCP1 (1.5%), CCT4 (0.9%), CCT7 (0.7%), and CCT8 (1.2%) (Fig. 3A). It is noteworthy that gene amplification and alteration of CCTs were the most evident in LUAD among different tissue types of lung cancer (Fig. S3, Supplemental Digital Content, http://links.lww.com/MD/M702). Moreover, it was observed that LUAD patients harboring CCTs mutations had a shorter OS than those without mutations (Fig. 3B). However, DFS exhibited no difference between the 2 groups (Fig. 3B). Promoter methylation of CCTs in LUAD was also assessed. Data from the UALCAN database indicated that CCT4, CCT5, CCT6A, and CCT8 exhibited significantly higher promoter methylation in normal tissues than in tumor tissues (Fig. 3C), suggesting that low promoter methylation may be responsible for upregulating these CCTs in LUAD.

### 3.4. Clinical significance of CCTs in LUAD

**Figure 4.**
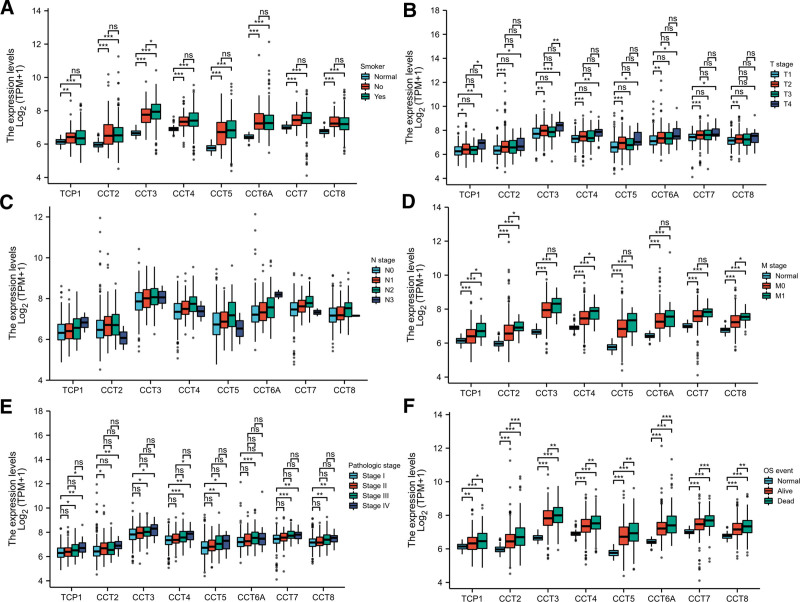
The association between CCTs expression and clinicopathological features of LUAD. The expression of CCTs was evaluated according to smoke status (A), T stage (B), N stage (C), M stage (D), pathological stage (E), and OS event (F). *P < .05, **P < .01, ***P < .001. CCTs = chaperonin-containing TCP-1 (T-complex protein 1) subunit genes, LUAD = lung adenocarcinoma.

Due to the abnormally high expression of CCTs in LUAD, the clinical implications of CCTs from TCGA data were studied further. In LUAD patients, no statistical difference was observed in the expression of CCTs between smokers and nonsmokers (Fig. 4A). All CCTs, except CCT8, exhibited significantly higher expression levels in LUAD patients with tumor stage IV as compared with stage I (Fig. 4B). However, no significant difference was observed in CCTs expression in LUAD patients in the N0, N1, or N2 stages (Fig. 4C). Patients who had distant metastasis demonstrated a higher probability of having increased expression of CCTs. However, this correlation was not statistically significant for the expression levels of CCT3, CCT5, CCT6A, and CCT7 (Fig. 4D). The correlation between the pathological stage and CCTs expression was also assessed. Most CCTs exhibited increased expression in stage III and IV patients compared with stage I patients (Fig. 4E). The association between CCTs expression and the survival of LUAD patients was also evaluated. Regarding the OS event, significantly higher CCTs expression was observed in deceased patients than in alive patients (Fig. 4F). The chi-square test also showed that the expression of most CCTs was strongly correlated with larger tumor size, distant metastasis, and lymph node metastasis (Table S3, Supplemental Digital Content, http://links.lww.com/MD/M710). These results indicate that CCTs upregulation in LUAD is strongly associated with advanced diseases of LUAD.

### 3.5. CCTs serve as diagnostic and prognostic indicators of LUAD

**Figure 5.**
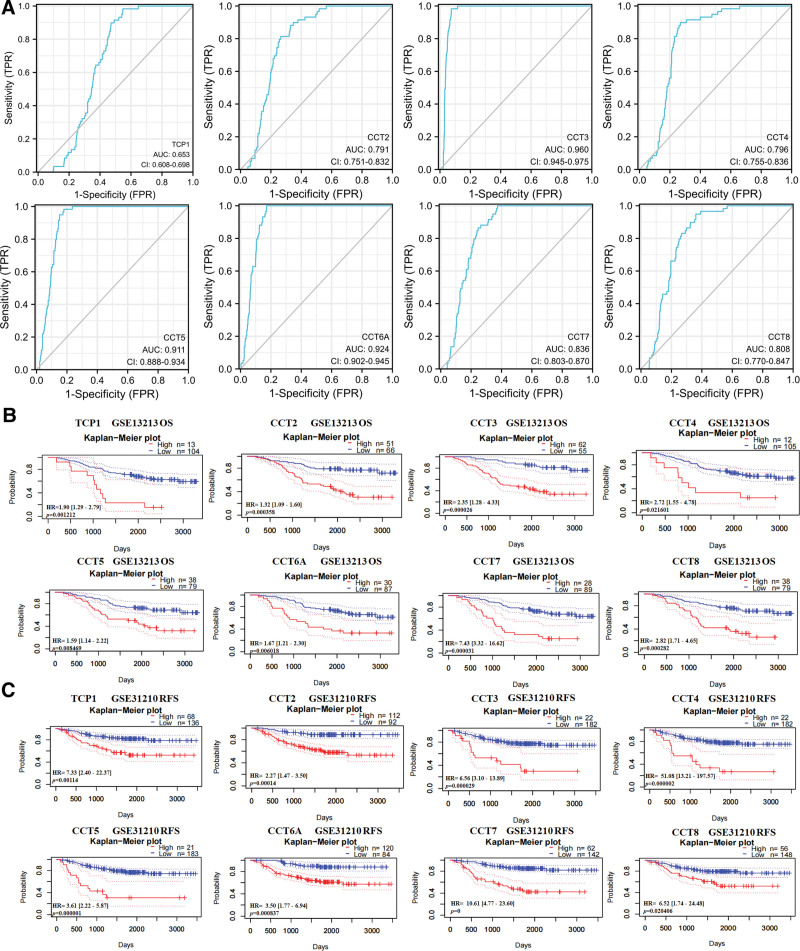
The diagnostic and prognostic value of CCTs in LUAD. (A) ROC curve of CCTs mRNA expression in LUAD cohort from TCGA database. Survival curves using the PrognoScan database are shown for OS (B) and RFS (C). CCTs = chaperonin-containing TCP-1 (T-complex protein 1) subunit genes, LUAD = lung adenocarcinoma, OS = overall survival, TCGA = The Cancer Genome Atlas.

**Figure 6.**
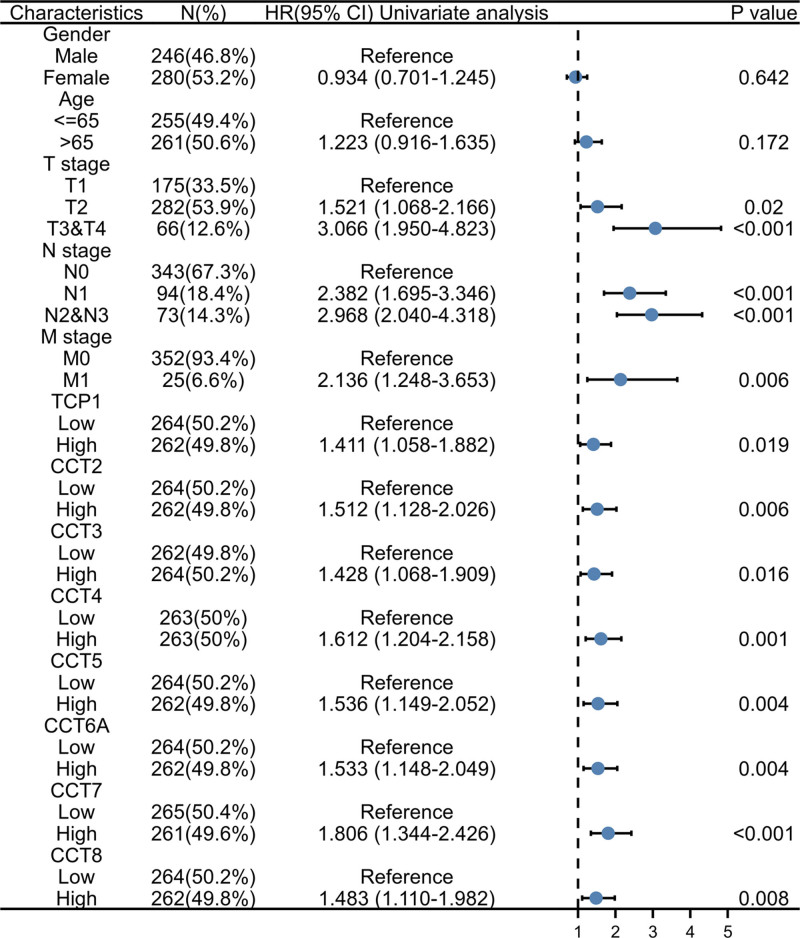
The univariate Cox analysis of the correlation of CCTs expression with OS from the TCGA database. CCTs = chaperonin-containing TCP-1 (T-complex protein 1) subunit genes, TCGA = The Cancer Genome Atlas.

Subsequently, the overexpression of CCTs in LUAD enabled the identification of the potential diagnostic utility of CCTs. According to the TCGA data, CCT3 (AUC = 0.960), CCT5 (AUC = 0.911), and CCT6A (AUC = 0.924) may serve as excellent biomarkers for the diagnosis of LUAD (Fig. 5A). Due to the correlation between CCTs and LUAD progression, further investigation was conducted to determine whether altered CCTs expression predicted poor outcome in LUAD patients. According to the PrognoScan database, LUAD patients with low CCTs expression had significantly longer OS and relapse-free survival than those with high CCTs expression (Fig. 5B and C). These results were validated using the GEPIA2.0 database (Fig. S4, Supplemental Digital Content, http://links.lww.com/MD/M703). The survival curves indicated that the expression of CCTs was associated with poor outcomes among patients with LUAD. Subsequently, univariate analysis revealed that the T, N, and M stages demonstrated a correlation between high CCTs expression and the OS of LUAD patients (Fig. 6). However, according to the multivariate analysis, CCTs expression was not an independent risk factor for OS in LUAD (Fig. S5, Supplemental Digital Content, http://links.lww.com/MD/M704).

### 3.6. Correlation between the expression levels of CCTs in LUAD

**Figure 7.**
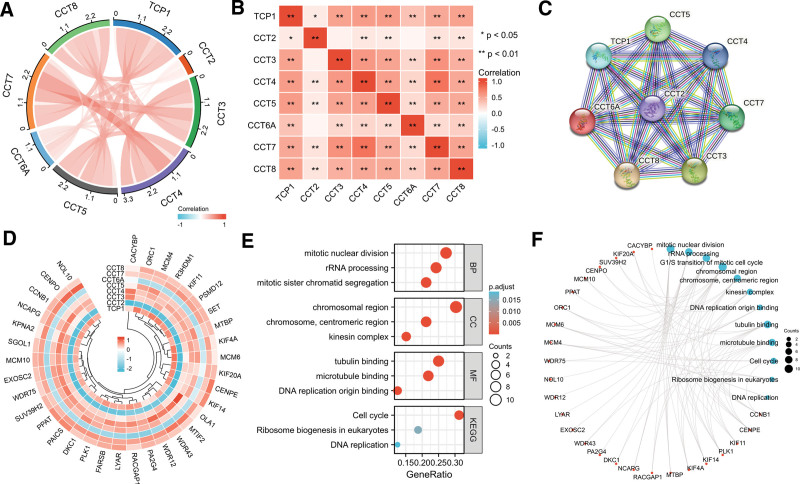
The association and correlation of genes among the CCTs. (A) Expression correlation among CCTs in the TCGA cohort. (B) Pearson correlation analysis of the CCTs expression in LUAD based on the GEPIA2.0 database. (C) PPI network of CCTs using STRING tool. (D) Correlation between CCTs and their co-expressed genes. (E, F) GO and KEGG pathway enrichment analysis of CCTs-co-expressed genes. *P < .05, **P < .01. CCTs = chaperonin-containing TCP-1 (T-complex protein 1) subunit genes, GO = gene ontology, KEGG = Kyoto Encyclopedia of Genes and Genome, LUAD = lung adenocarcinoma, PPI = protein–protein interaction.

Due to the comparable clinical significance of CCTs in LUAD, further investigation was conducted to observe whether the co-expression of CCTs exerts oncogenic roles in LUAD. The expression correlation data of the CCTs in LUAD were obtained from the GEPIA2.0 database (Fig. S6, Supplemental Digital Content, http://links.lww.com/MD/M705). The overall analysis indicated that TCP1, CCT3, CCT4, CCT5, CCT7, and CCT8 expression was positively and significantly associated with each other (Fig. 7A and B). However, CCT2 and CCT6A expression did not significantly correlate with other CCTs (Fig. 7A and B), suggesting that CCT2 and CCT6A promote LUAD progression in a manner distinct from that of other CCTs. The protein interactions, including all CCTs, were investigated using the STRING tool, and close interactions between these CCTs were observed (Fig. 7C).

### 3.7. Functional enrichment analysis of CCTs-correlated genes

A comprehensive investigation of the molecular mechanism underlying the tumor-promoting properties of CCTs was performed. CCTs expression-correlated proteins were obtained from the GEPIA2.0 database, except for CCT2 and CCT6A, whose functions in LUAD may be accounted for by alternative mechanisms. A total of 35 proteins exhibited correlations in expression with TCP1, CCT3, CCT4, CCT5, CCT7, and CCT8 (Fig. S7, Supplemental Digital Content, http://links.lww.com/MD/M706). The respective correlations were obtained using the GEPIA2.0 database and visualized in a loop heatmap (Fig. 7D). The CCTs, except for CCT2 and CCT6A, exhibited a significantly close association with the 35 proteins (Fig. 7D). The 35 co-expressed genes of CCTs were subjected to gene ontology and Kyoto Encyclopedia of Genes and Genome analysis to better understand the potential involvement of CCTs in LUAD. CCT-co-expressed genes were involved in the mitotic nuclear division, chromosomal region, tubulin binding, and cell cycle (Fig. 7E and F).

### 3.8. Analysis of CCTs-related Hub genes through protein–protein interaction network

**Figure 8.**
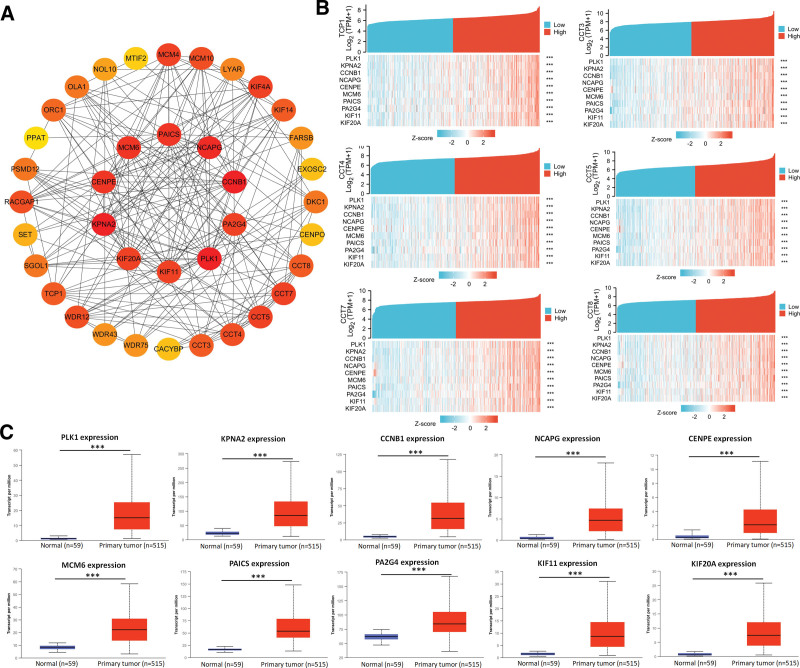
Analysis of CCTs-related Hub genes. (A) Hub genes were shown in the PPI network using the Cytoscape tool. (B) The expression of the Hub genes in LUAD patients with low and high CCTs expression groups using TCGA data. (C) The expression of the Hub genes was measured based on the UALCAN database. ***P < .001. CCTs = chaperonin-containing TCP-1 (T-complex protein 1) subunit genes, LUAD = lung adenocarcinoma, TCGA = The Cancer Genome Atlas.

The Cytoscape tool was utilized to analyze protein-protein interaction network analysis which showed that the 10 representative Hub genes (PLK1, KPNA2, CCNB1, NCAPG, CENPE, MCM6, PAICS, PA2G4, KIF11, and KIF20A) were closely related to CCTs expression and function (Fig. 8A). The association between the top 10 Hub genes and CCTs, except for CCT2 and CCT6A, is shown in the form of a heatmap (Fig. 8B). Additionally, the expression and survival of the 10 Hub genes were evaluated. According to these findings, tumor tissues had significantly higher levels of the Hub genes than normal tissues (Fig. 8C). Notably, patients with LUAD who had elevated expression of all these Hub genes had a poor prognosis (Fig. S8, Supplemental Digital Content, http://links.lww.com/MD/M707).

### 3.9. CCTs are associated with Th2 immune cell infiltration

**Figure 9.**
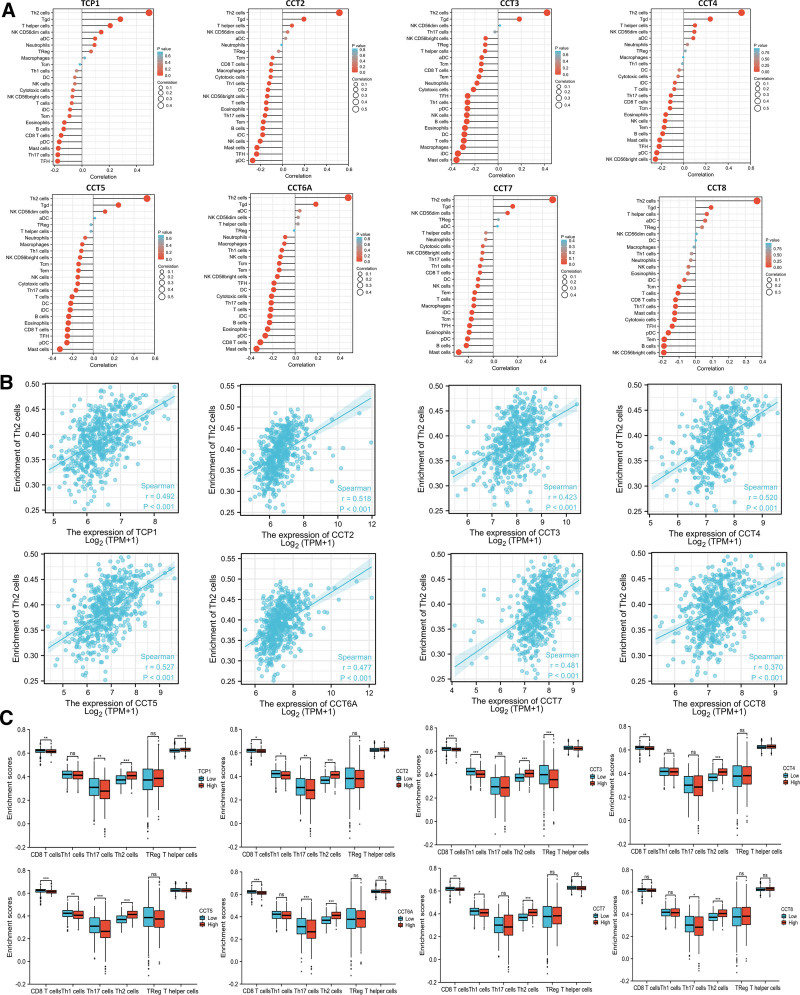
Correlation analysis of CCTs expression with TILs. (A) The correlation between CCTs expression and TILs was analyzed based on TCGA data. (B) The correlation between CCTs expression and Th2 cell infiltration was analyzed based on TCGA data. (C) Enrichment of CD8^+^ T, Th1, Th17, Th2, Treg, and T helper cells in low and high CCTs expression LUAD tumor tissues. *P < .05, **P < .01, ***P < .001. CCTs = chaperonin-containing TCP-1 (T-complex protein 1) subunit genes, TCGA = The Cancer Genome Atlas, TILs = tumor-infiltrating lymphocytes, LUAD = lung adenocarcinoma.

Tumor progression is associated with the tumor microenvironment, particularly with immune cell infiltration. Thus, the relationship between CCTs expression and tumor-infiltrating lymphocytes was investigated further. Based on TCGA data, it was observed that the expression of all CCTs was positively correlated with the infiltration of Th2 cells (Fig. 9A and B). TCP1 and CCT2 exhibited a negative association with T follicular helper-like cells and plasmacytoid dendritic cells, respectively. Additionally, CCT3, CCT5, CCT6A, and CCT7 were negatively correlated with mast cells. A negative correlation was observed between the expression of CCT4 and CCT8 and the infiltration of CD56-bright NK cells (Fig. 9A).

**Figure 10.**
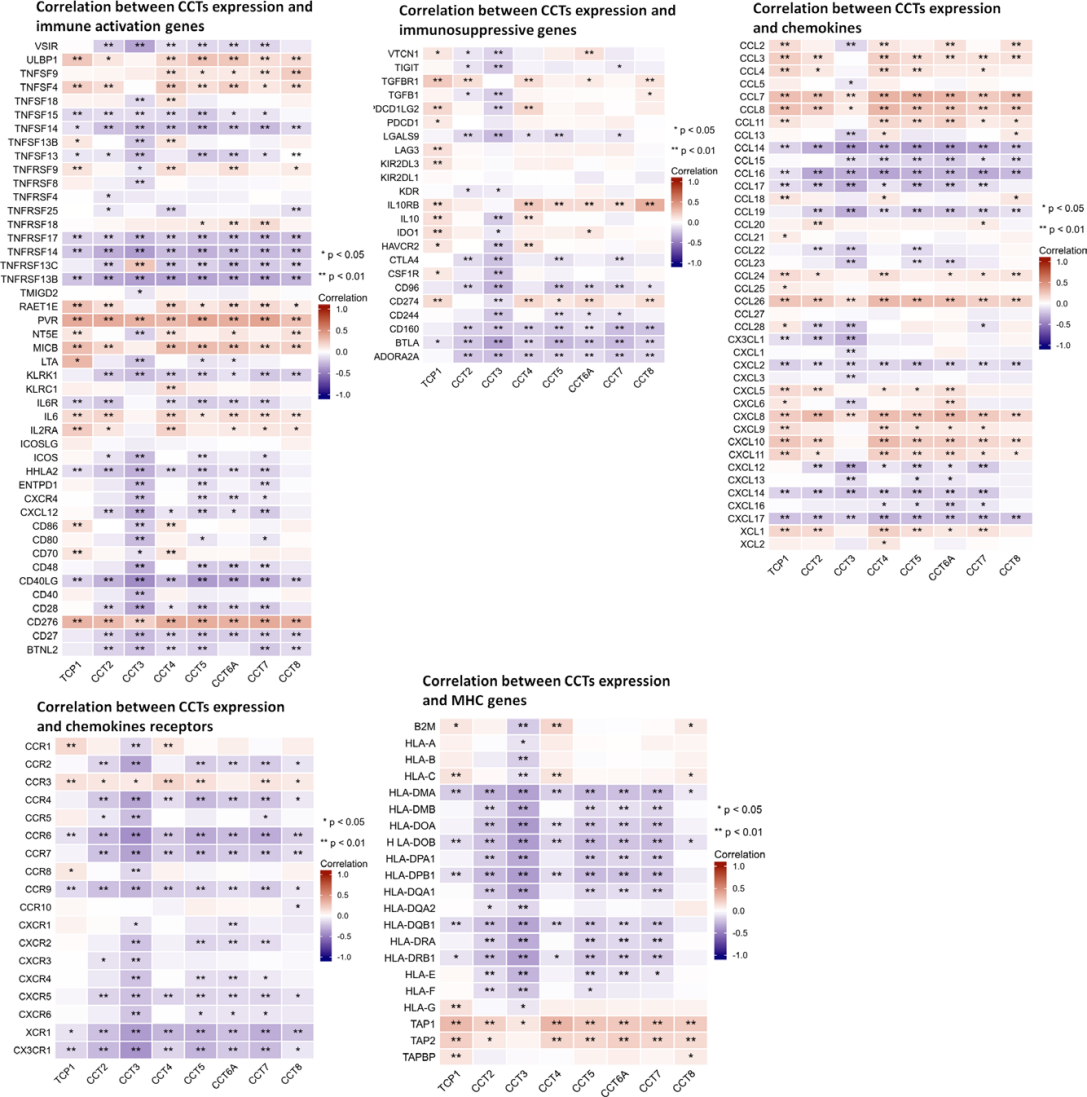
Co-expression of CCTs and immune-related genes. *P < .05, **P < .01, ***P < .001. CCTs = chaperonin-containing TCP-1 (T-complex protein 1) subunit genes.

Tumor-specific T cells significantly affect the clinical outcomes of patients with cancer. The expression levels of CD8^+^ T, T helper (Th) type 1 (Th1), Th17, Th2, Treg, and T helper cells was evaluated in low and high CCTs expression LUAD tumor tissues. Figure 9C reveals that all tissues with high CCTs expression exhibited greater Th2 cell infiltration than those with low CCTs expression. Additionally, tissues with high CCT2, CCT3, CCT5, and CCT7 had decreased Th1 cells (Fig. 9C). Notably, LUAD tissues with high expression of CCTs, except for CCT8, demonstrated reduced CD8^+^ T cell infiltration (Fig. 9C). These findings indicated that tumor-promoting role of CCTs is linked to the involvement of increased Th2 cells and decreased Th1 and CD8^+^ T cells. Furthermore, gene correlation analysis was conducted to investigate the associations between CCTs expression and immune-related genes, such as immune activation, immunosuppressive, chemokine, chemokine receptors, and MHC genes. The results indicated that most immune-related genes were positively or negatively co-expressed with CCTs (Fig. 10).

## 4. Discussion

Due to its high incidence rate and mortality, LUAD presents a constant challenge to clinical physicians and researchers in terms of precise diagnosis and efficient therapy. Therefore, it is critical to investigate more precise and sensitive biomarkers for the early identification of LUAD and personalized treatment. In this study, a comprehensive analysis of CCTs in LUAD was conducted using TCGA and other public databases such as UALCAN, GEPIA 2.0, TIMER 2.0, PrognoScan, cBioPortal, GEO, and STRING. The TCGA database was established in 2006 and compiled gene expression and clinical data on 33 different tumor types, ranging from hematological to solid tumors and from mildly to severely aggressive tumors.^[28]^ Considerable progress has been achieved using TCGA to identify novel oncogenic biomarkers and establish molecular subtypes.^[29–31]^ Many other platforms, such as UALCAN and GEPIA 2.0, have been developed to identify CCT family genes that may serve as novel and useful biomarkers for the diagnosis of LUAD and targets for LUAD treatment.

Numerous studies have examined the role of CCTs, such as CCT3, CCT5, and CCT6A, in lung cancer, particularly NSCLC. Upregulation of CCT3 levels was observed in NSCLC tumor tissues, which was linked to an unfavorable patient prognosis.^[32]^ Another study revealed that cisplatin-resistant A549 cells exhibited higher expression of CCT3 than cisplatin-sensitive A549 cells, suggesting that CCT3 may enhance cisplatin resistance by triggering the JAK2/STAT3 pathway.^[33]^ In CCT3-deficient LUAD cells, glycolysis was inhibited, and ATP production was reduced, resulting in decreased cell growth and metastasis.^[34]^ CCT6A expression was linked to the poor prognosis of LUAD patients and was coamplifying and co-expressing with EGFR.^[35]^ Interestingly, anti-CCT5 auto-antibody was present in 51% of NSCLC patients but only in 2.5% of healthy controls, indicating that CCT5 is a tumor-associated antigen that can serve as a diagnostic marker for NSCLC.^[36]^ Given the gradually emerging role of CCTs in NSCLC, it is crucial to develop a comprehensive understanding of CCTs to facilitate further research. Our study showed that CCTs, except CCT6B, had higher expression levels in NSCLC tumor tissues, such as LUAD and LUSC. However, high expression levels of all CCTs (except CCT6B) predicted an unfavorable outcome in LUAD patients compared with LUSC patients. Thus, CCT subunits may serve as prognostic biomarkers, specifically in LUAD rather than LUSC.

Chaperones are initially characterized by their ability to interact with other proteins and assist in the folding of substrate proteins.^[37]^ The most extensively studied chaperones are heat shock proteins (HSPs), which have multiple clinical implications as biomarkers for cancer diagnosis and anti-cancer treatment.^[38]^ CCTs are another novel family of molecular chaperones that require further study. Cytoskeleton proteins, such as actin and tubulin, are known substrates of CCTs.^[39]^ According to one study, deletion of CCT8 suppressed the formation of nuclear actin filaments in T cells, affecting T cell maturation and function.^[40]^ Furthermore, CCT8 influenced the migration and invasion of ESCC cells by regulating the expression of α-actin and β-tubulin in esophageal squamous cell carcinoma.^[16]^ Additionally, interactions between CCTs and other functional proteins were reported. CCT3 interacts with the β-strand rich and DNA-binding domain of Stat3, contributing to its biosynthesis and transcriptional activity.^[41]^ CCTs were verified to bind to Cdc20, and their presence was essential for the Cdc20-dependent cell cycle process.^[42]^ In metastatic cancer cells, the CCT complex directly bound and stabilized XIAP and β-catenin expression, facilitating chemoresistance and metastasis.^[14]^ CCT3 interacts with eukaryotic translation initiation factor 3, affecting cytoplasmic translation in LUAD.^[34]^ Moreover, CCTs interacted with PLK1,^[43]^ cyclinE,^[44]^ cyclinD,^[45]^ and p53,^[46]^ which were closely related to cancer progression. In this study, screening of CCTs-related Hub genes revealed that they were upregulated in LUAD patients and predicted poor outcomes. Additional research is required to verify whether these genes can serve as substrates for CCTs in LUAD.

The tumor microenvironment comprises a variety of heterogeneous cells, including immune cells, endothelial cells, and fibroblasts. Avoiding immune destruction continues to be an important hallmark of cancer.^[47]^ Continuous interactions between the tumor microenvironment and cancer cells promote tumor progression and metastasis and affect therapeutic response and clinical outcome.^[48,49]^ T cell-mediated immune responses have attracted widespread interest and have been used as therapeutic targets in diverse pre-clinical and clinical models.^[50,51]^ Th1 and Th2 cells are the two classes of CD4^+^ T cells that were first studied and were characterized by the production of interferon-gamma and interleukin-4, respectively.^[52]^ Cancer patients undergo a dominant Th2 differentiation state, where the cytokines produced by Th2 cells have an inhibitory effect on the proliferation and differentiation of Th1 cells, impairing the function of cytotoxic T cells and weakening the body’s immunity against tumors.^[50]^ In this study, a strong correlation was observed between Th2 cell infiltration and CCT expression in LUAD. These data were in agreement with those of a previous study that indicated that CCT8 expression is necessary for T cell metabolism and Th2 cell polarization. The previous study used a CCT8 KO mouse model and determined that CCT8 is essential for the protective immunity of the body against intestinal helminths.^[40]^ Thus, CCTs may be essential for T cells to be Th2 polarized, weakening the immune response to tumor cells.

HSPs have been widely studied as protein chaperones, and highly selective and specific inhibitors of HSPs have been developed.^[53–55]^ Furthermore, some inhibitors have been subjected to clinical evaluation. Due to the significance of CCTs in cancer, researchers have also attempted to identify inhibitors targeting CCTs. A compound was reported to impede the interaction between CCT2 and β-tubulin, thereby inhibiting the migration and invasion of LUAD cells.^[17]^ Furthermore, a different drug called anticarin-β demonstrated encouraging anti-osteosarcoma activity by selectively inhibiting CCT4 to damage proteostasis.^[56]^ Anticarin-β could strongly decrease CCT4-mediated STAT3 maturation and had exceptional anti-tumor efficacy in both orthotopic and patient-derived xenograft models of osteosarcoma.^[56]^ Collectively, it is promising to target CCTs for cancer treatment, and more efforts are needed to develop CCT inhibitors.

In conclusion, our study encompassed the expression, mutations, methylation, clinicopathological traits, prognosis, associated genes, and functional enrichment of CCTs in LUAD using TCGA data and other freely accessible online bioinformatic platforms and tools. CCTs expression was elevated in LUAD tumor tissues, and elevated CCT levels were associated with severe disease and worse prognoses among LUAD patients. Additionally, Th2 cell infiltration in the tumor microenvironment and the mitosis-driven cell cycle process were both mediated by CCTs. Our research suggested that CCTs may serve as prognostic indicators and new targets for LUAD treatment.

## Acknowledgments

The authors would like to sincerely thank the TCGA for data sharing, as well as UALCAN, GEPIA2.0, TIMER2.0, PrognoScan, cBioPortal, GEO, and STRING for providing data processing and analysis.

## Author contributions

**Conceptualization:** Li Han, Hua Bian.

**Data curation:** Ruijuan Du, Zijun Zhou, Yunlong Huang.

**Formal analysis:** Kai Li, Kelei Guo.

**Funding acquisition:** Ruijuan Du.

**Methodology:** Zijun Zhou, Yunlong Huang.

**Project administration:** Li Han, Hua Bian.

**Supervision:** Li Han, Hua Bian.

**Writing – original draft:** Ruijuan Du.

**Writing – review & editing:** Kai Li, Kelei Guo.

## Supplementary Material







**Figure:**
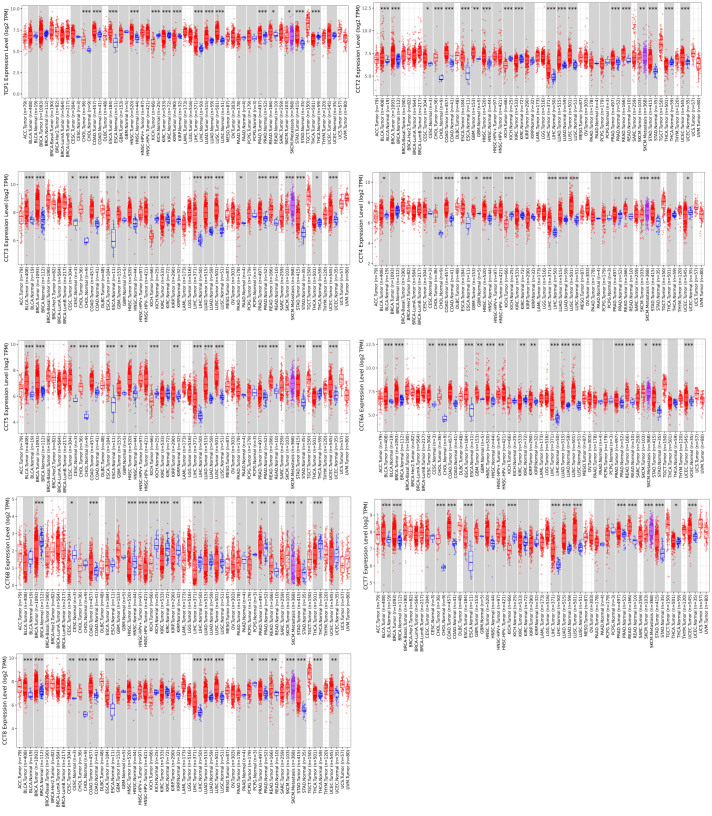


**Figure:**
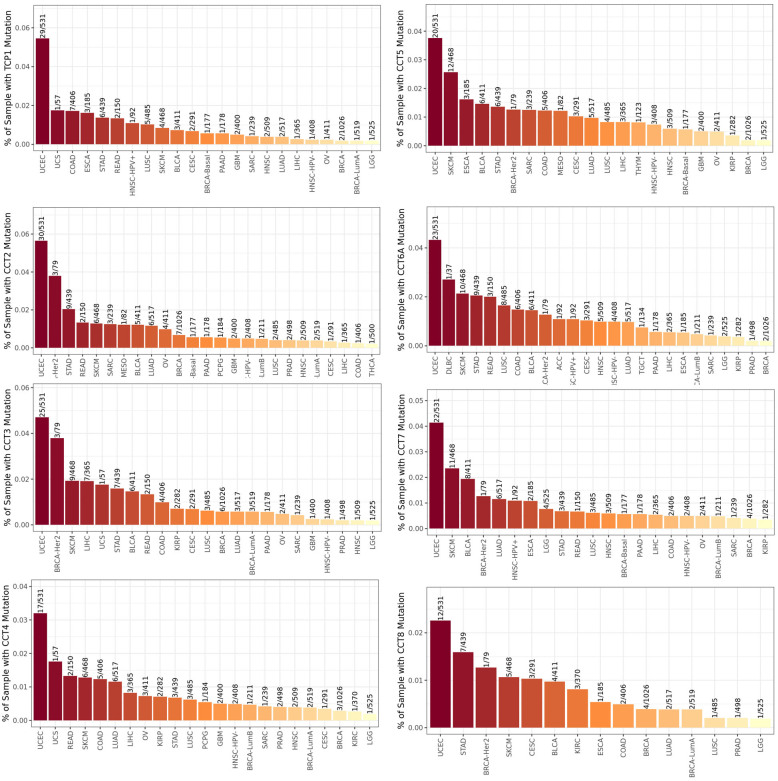


**Figure:**
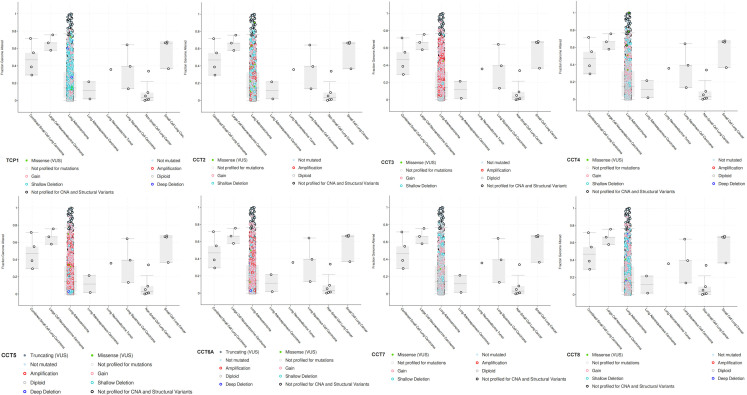


**Figure:**
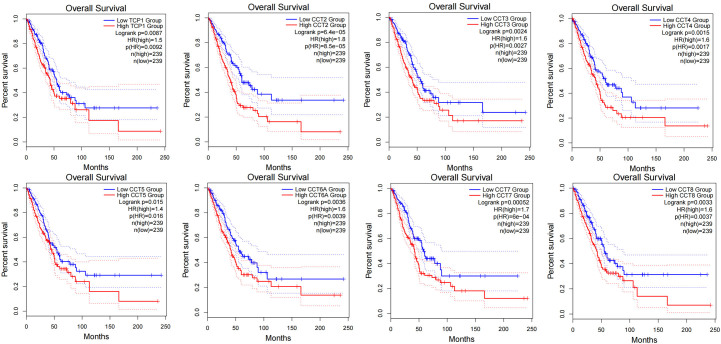


**Figure:**
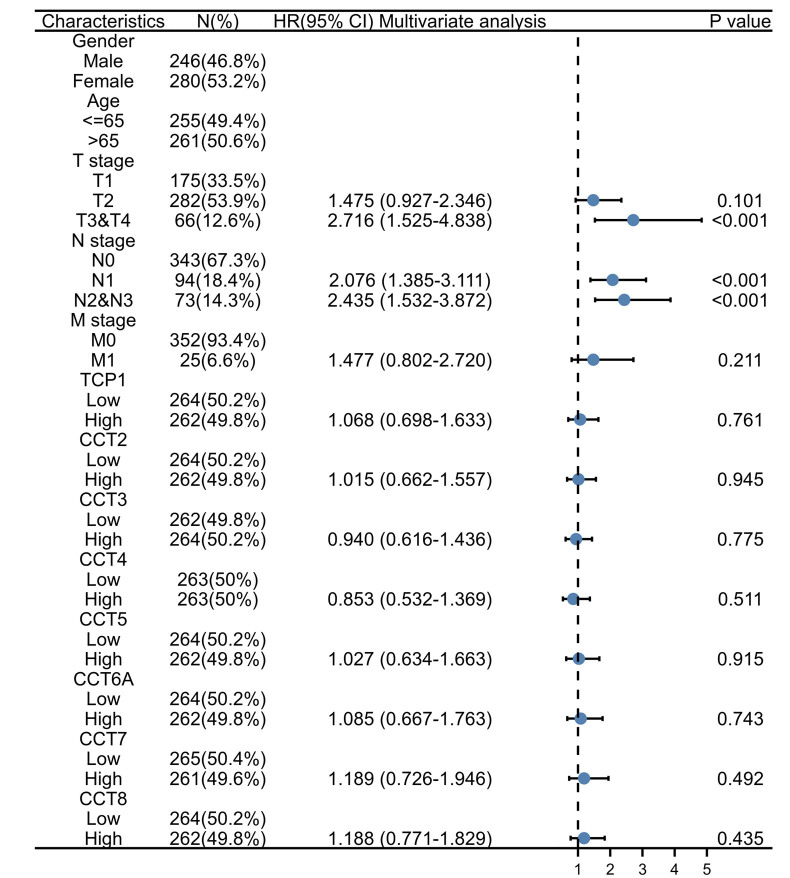


**Figure:**
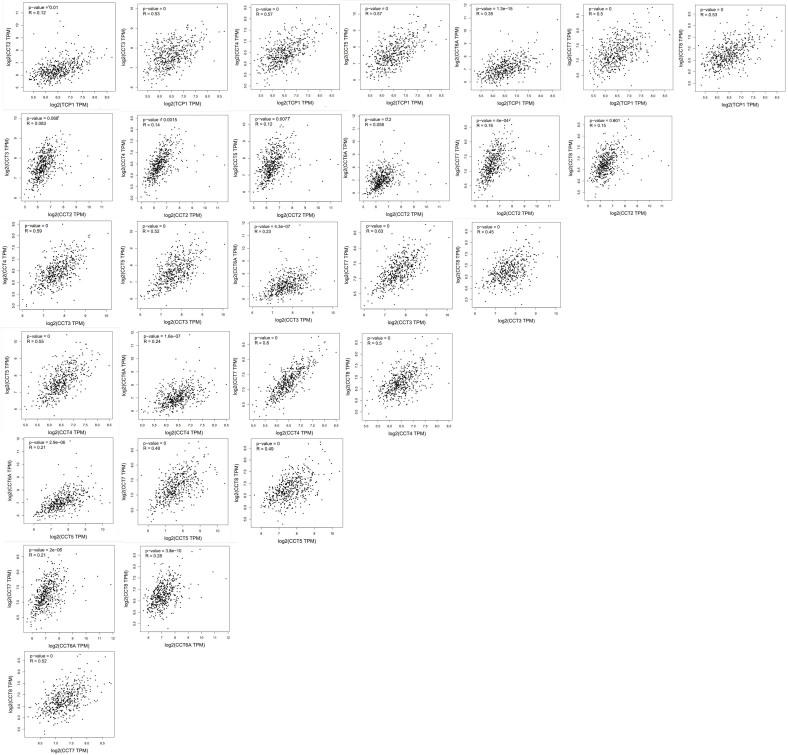


**Figure:**
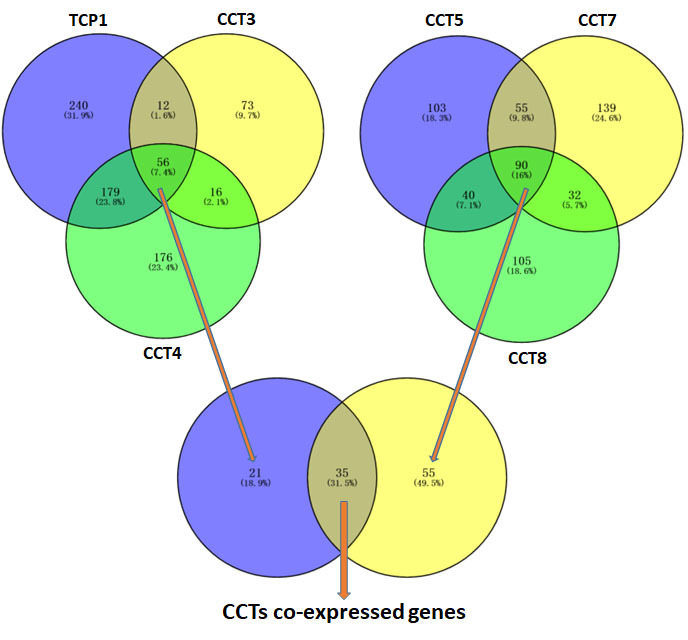


**Figure:**