# Supplementary material for: Chaperonin-containing TCP-1 subunit genes are potential prognostic biomarkers and are correlated with Th2 cell infiltration in lung adenocarcinoma: An observational study
Source: Medicine (Baltimore). 2024 May 31;103(22):e38387. doi: 10.1097/MD.0000000000038387 (PMC11142841; doi:10.1097/MD.0000000000038387)
Supplement: Supplementary file 1 [file medi-103-e38387-s002.docx]

Table S1. The expression of CCTs among various cancer types.

|  | TCP1 | CCT2 | CCT3 | CCT4 | CCT5 | CCT6A | CCT6B | CCT7 | CCT8 |
| --- | --- | --- | --- | --- | --- | --- | --- | --- | --- |
| BLCA | NS | *** | *** | * | *** | *** | NS | *** | *** |
| BRCA | NS | *** | *** | NS | *** | *** | *** | *** | *** |
| CESC | NS | * | * | NS | ** | ** | * | NS | ** |
| CHOL | *** | *** | *** | *** | *** | *** | NS | *** | *** |
| COAD | *** | *** | *** | *** | *** | *** | *** | *** | *** |
| ESCA | *** | *** | *** | *** | *** | *** | NS | *** | *** |
| GBM | NS | ** | * | * | * | ** | NS | NS | NS |
| HNSC | *** | *** | *** | *** | *** | *** | *** | *** | *** |
| KICH | *** | *** | *** | *** | *** | NS | *** | *** | *** |
| KIRC | *** | *** | *** | NS | NS | ** | *** | NS | NS |
| KIRP | *** | NS | NS | * | ** | ** | ** | NS | NS |
| LIHC | *** | *** | *** | *** | *** | *** | *** | *** | *** |
| LUAD | *** | *** | *** | *** | *** | *** | NS | *** | *** |
| LUSC | *** | *** | *** | *** | *** | *** | NS | *** | *** |
| PAAD | NS | NS | * | NS | NS | NS | NS | NS | NS |
| PCPG | NS | NS | NS | NS | NS | NS | * | NS | * |
| PRAD | *** | *** | *** | ** | *** | *** | ** | *** | *** |
| READ | * | *** | *** | *** | *** | *** | * | *** | ** |
| STAD | *** | *** | *** | *** | *** | *** | * | *** | *** |
| THCA | *** | *** | * | NS | *** | NS | *** | * | *** |
| UCEC | NS | *** | *** | * | *** | *** | NS | *** | ** |

Black asterisk means significant higher expression of CCTs in tumor tissues than normal tissues. Red asterisk means significant higher expression of CCTs in normal tissues than tumor tissues. **p* < 0.05, ***p* < 0.01, ****p* < 0.001.
