# Supplementary material for: Chaperonin-containing TCP-1 subunit genes are potential prognostic biomarkers and are correlated with Th2 cell infiltration in lung adenocarcinoma: An observational study
Source: Medicine (Baltimore). 2024 May 31;103(22):e38387. doi: 10.1097/MD.0000000000038387 (PMC11142841; doi:10.1097/MD.0000000000038387)
Supplement: Supplementary file 2 [file medi-103-e38387-s003.docx]

Table S2. Correlation between CCTs expression and patients survival in pan-cancer analysis.

|  | TCP1 | CTT2 | CTT3 | CTT4 | CTT5 | CTT6A | CCT6B | CTT7 | CTT8 |
| --- | --- | --- | --- | --- | --- | --- | --- | --- | --- |
| BLCA | NS | NS | NS | NS | NS | NS | NS | NS | NS |
| BRCA | *** | * | NS | ** | ** | ** | NS | ** | ** |
| CESC | * | NS | ** | NS | NS | NS | NS | NS | NS |
| CHOL | NS | NS | NS | NS | NS | NS | NS | NS | NS |
| COAD | NS | NS | NS | NS | NS | ** | NS | NS | NS |
| ESCA | * | NS | NS | * | NS | NS | NS | NS | NS |
| GBM | NS | NS | NS | NS | NS | NS | NS | NS | NS |
| HNSC | NS | * | * | NS | * | * | NS | * | NS |
| KICH | * | * | NS | NS | NS | NS | NS | NS | NS |
| KIRC | * | NS | NS | * | NS | NS | ** | * | ** |
| KIRP | NS | NS | * | NS | NS | * | ** | NS | NS |
| LIHC | ** | *** | *** | *** | * | ** | ** | *** | * |
| LUAD | ** | *** | ** | ** | * | ** | NS | *** | ** |
| LUSC | NS | NS | NS | NS | NS | NS | NS | NS | NS |
| PAAD | NS | NS | NS | NS | NS | NS | NS | NS | NS |
| PCPG | NS | NS | NS | NS | NS | NS | NS | NS | NS |
| PRAD | NS | NS | NS | NS | NS | NS | NS | NS | NS |
| READ | NS | NS | NS | NS | NS | NS | NS | NS | NS |
| STAD | NS | NS | NS | NS | NS | NS | NS | NS | NS |
| THCA | NS | NS | NS | NS | NS | NS | * | NS | NS |
| UCEC | NS | ** | NS | NS | NS | NS | * | NS | NS |

Black asterisk means high CCTs expression predicted poorer patients outcome. Red asterisk means high CCTs expression predicted longer survival time. **p* < 0.05, ***p* < 0.01, ****p* < 0.001.
