# Supplementary material for: Chaperonin-containing TCP-1 subunit genes are potential prognostic biomarkers and are correlated with Th2 cell infiltration in lung adenocarcinoma: An observational study
Source: Medicine (Baltimore). 2024 May 31;103(22):e38387. doi: 10.1097/MD.0000000000038387 (PMC11142841; doi:10.1097/MD.0000000000038387)
Supplement: Supplementary file 3 [file medi-103-e38387-s006.docx]

Table S3.The relationships between CCTs expression and TNM stage features in LUAD patients.

|  | **TCP1** | | |  | **CCT2** | | |  | **CCT3** | | |  | **CCT4** | | |
| --- | --- | --- | --- | --- | --- | --- | --- | --- | --- | --- | --- | --- | --- | --- | --- |
| Characteristic | Low | High | p |  | Low | High | p |  | Low | High | p |  | Low | High | p |
| n | 267 | 268 |  |  | 267 | 268 |  |  | 267 | 268 |  |  | 267 | 268 |  |
| T stage,  n (%) |  |  | **0.003** |  |  |  | **0.002** |  |  |  | **< 0.001** |  |  |  | **< 0.001** |
| T1 | 104 (19.5%) | 71 (13.3%) |  |  | 108 (20.3%) | 67 (12.6%) |  |  | 106 (19.9%) | 69 (13%) |  |  | 111 (20.9%) | 64 (12%) |  |
| T2 | 135 (25.4%) | 154 (28.9%) |  |  | 126 (23.7%) | 163 (30.6%) |  |  | 130 (24.4%) | 159 (29.9%) |  |  | 125 (23.5%) | 164 (30.8%) |  |
| T3 | 24 (4.5%) | 25 (4.7%) |  |  | 23 (4.3%) | 26 (4.9%) |  |  | 25 (4.7%) | 24 (4.5%) |  |  | 25 (4.7%) | 24 (4.5%) |  |
| T4 | 4 (0.8%) | 15 (2.8%) |  |  | 8 (1.5%) | 11 (2.1%) |  |  | 4 (0.8%) | 15 (2.8%) |  |  | 4 (0.8%) | 15 (2.8%) |  |
| N stage,  n (%) |  |  | **0.021** |  |  |  | **0.014** |  |  |  | 0.469 |  |  |  | **< 0.001** |
| N0 | 187 (36%) | 161 (31%) |  |  | 187 (36%) | 161 (31%) |  |  | 179 (34.5%) | 169 (32.6%) |  |  | 193 (37.2%) | 155 (29.9%) |  |
| N1 | 42 (8.1%) | 53 (10.2%) |  |  | 36 (6.9%) | 59 (11.4%) |  |  | 43 (8.3%) | 52 (10%) |  |  | 40 (7.7%) | 55 (10.6%) |  |
| N2 | 27 (5.2%) | 47 (9.1%) |  |  | 31 (6%) | 43 (8.3%) |  |  | 32 (6.2%) | 42 (8.1%) |  |  | 22 (4.2%) | 52 (10%) |  |
| N3 | 1 (0.2%) | 1 (0.2%) |  |  | 1 (0.2%) | 1 (0.2%) |  |  | 1 (0.2%) | 1 (0.2%) |  |  | 1 (0.2%) | 1 (0.2%) |  |
| M stage,  n (%) |  |  | **0.039** |  |  |  | **0.007** |  |  |  | 0.095 |  |  |  | **0.047** |
| M0 | 171 (44.3%) | 190 (49.2%) |  |  | 165 (42.7%) | 196 (50.8%) |  |  | 171 (44.3%) | 190 (49.2%) |  |  | 168 (43.5%) | 193 (50%) |  |
| M1 | 6 (1.6%) | 19 (4.9%) |  |  | 4 (1%) | 21 (5.4%) |  |  | 7 (1.8%) | 18 (4.7%) |  |  | 6 (1.6%) | 19 (4.9%) |  |

Continued

|  | **CCT5** | | |  | **CCT6A** | | |  | **CCT7** | | |  | **CCT8** | | |
| --- | --- | --- | --- | --- | --- | --- | --- | --- | --- | --- | --- | --- | --- | --- | --- |
| Characteristic | Low | High | p |  | Low | High | p |  | Low | High | p |  | Low | High | p |
| n | 267 | 268 |  |  | 267 | 268 |  |  | 267 | 268 |  |  | 267 | 268 |  |
| T stage,  n (%) |  |  | **< 0.001** |  |  |  | **0.003** |  |  |  | **0.002** |  |  |  | **0.004** |
| T1 | 112 (21.1%) | 63 (11.8%) |  |  | 105 (19.7%) | 70 (13.2%) |  |  | 107 (20.1%) | 68 (12.8%) |  |  | 106 (19.9%) | 69 (13%) |  |
| T2 | 123 (23.1%) | 166 (31.2%) |  |  | 132 (24.8%) | 157 (29.5%) |  |  | 130 (24.4%) | 159 (29.9%) |  |  | 130 (24.4%) | 159 (29.9%) |  |
| T3 | 25 (4.7%) | 24 (4.5%) |  |  | 23 (4.3%) | 26 (4.9%) |  |  | 20 (3.8%) | 29 (5.5%) |  |  | 23 (4.3%) | 26 (4.9%) |  |
| T4 | 5 (0.9%) | 14 (2.6%) |  |  | 5 (0.9%) | 14 (2.6%) |  |  | 7 (1.3%) | 12 (2.3%) |  |  | 6 (1.1%) | 13 (2.4%) |  |
| N stage,  n (%) |  |  | **< 0.001** |  |  |  | **< 0.001** |  |  |  | **< 0.001** |  |  |  | **< 0.001** |
| N0 | 190 (36.6%) | 158 (30.4%) |  |  | 190 (36.6%) | 158 (30.4%) |  |  | 197 (38%) | 151 (29.1%) |  |  | 183 (35.3%) | 165 (31.8%) |  |
| N1 | 42 (8.1%) | 53 (10.2%) |  |  | 43 (8.3%) | 52 (10%) |  |  | 38 (7.3%) | 57 (11%) |  |  | 49 (9.4%) | 46 (8.9%) |  |
| N2 | 22 (4.2%) | 52 (10%) |  |  | 23 (4.4%) | 51 (9.8%) |  |  | 16 (3.1%) | 58 (11.2%) |  |  | 21 (4%) | 53 (10.2%) |  |
| N3 | 1 (0.2%) | 1 (0.2%) |  |  | 0 (0%) | 2 (0.4%) |  |  | 2 (0.4%) | 0 (0%) |  |  | 2 (0.4%) | 0 (0%) |  |
| M stage,  n (%) |  |  | 0.330 |  |  |  | 0.455 |  |  |  | **0.042** |  |  |  | **0.035** |
| M0 | 174 (45.1%) | 187 (48.4%) |  |  | 180 (46.6%) | 181 (46.9%) |  |  | 170 (44%) | 191 (49.5%) |  |  | 173 (44.8%) | 188 (48.7%) |  |
| M1 | 9 (2.3%) | 16 (4.1%) |  |  | 10 (2.6%) | 15 (3.9%) |  |  | 6 (1.6%) | 19 (4.9%) |  |  | 6 (1.6%) | 19 (4.9%) |  |
